# Cervical Pediatric Spine Trauma Managed With Open Spinal Fixation and Instrumentation and a Review of the Literature

**DOI:** 10.7759/cureus.14004

**Published:** 2021-03-19

**Authors:** Michael J Gigliotti, Noa Farou, Sandip Salyvia, John Kelleher, Elias Rizk

**Affiliations:** 1 Neurological Surgery, Penn State Health Milton S. Hershey Medical Center, Hershey, USA; 2 Medicine, College of Medicine, Penn State Health Milton S. Hershey Medical Center, Hershey, USA; 3 Neurosurgery, Penn State Health Milton S. Hershey Medical Center, Hershey, USA; 4 Neurosurgery, Penn State Health, Hershey, USA

**Keywords:** accidental trauma, fusion, cervical, spinal injury, trauma

## Abstract

Cervical spine injuries in the pediatric population are rare. Most injuries to the cervical spinal cord and vertebral column can be managed nonoperatively; however, surgical management may be required in certain clinical scenarios. A posterior surgical approach has been previously preferred; however, the utilization of anterior spinal fixation and instrumentation has been limited. We present a small case series of patients presenting with a traumatic cervical spine injury and detail the feasibility of craniocervical junction (CVJ) and subaxial spinal fixation in the pediatric population.

We report four cases involving pediatric patients, all of whom presented with cervical spine injuries necessitating operative intervention using a combination of the anterior and posterior operative approaches. All four patients recovered well, did not require surgical revision, and were neurologically intact at the last follow-up.

Therefore, we conclude that spinal arthrodesis is a safe, effective way to manage spinal injuries in the cervical spine following traumatic injury.

## Introduction

Cervical spine injuries in children are rare, occurring in 1% to 2% of the pediatric population [1-2]. Motor vehicle accidents account for the most frequent cause of cervical spine injury, followed by pedestrian injuries, falls, and sports injuries [3]. The craniovertebral junction is the most susceptible zone of pediatric spine injury and potential instability due to the predisposition to increased motion in children, less-developed muscles and ligaments, and an unfused dentocentral synchondrosis prior to eight years of age [4]. Similarly, the upper cervical spine is predisposed to injury from flexion forces due to hypermobility secondary to ligamentous laxity, particularly between C2 and C3 [4]. Although children are more likely to present with upper cervical injury the younger they are, disparities between upper and lower cervical spinal injury rates dissipate by adolescence [4].

Most injuries to the cervical spinal cord and vertebral column can be managed nonoperatively with various external stabilization devices. Still, they may require operative management based on the stability of the injury [4-5]. Historically, a posterior approach has been preferred due to damage to the superior and inferior vertebral endplates resulting in spinal instability and deformity [5]. However, the modern operative approach to these injuries depends on the site of injury and compression, patient age, size of bony elements, and surgeon experience [4].

Evidence supporting the utility of anterior spinal instrumentation and fusion is rare and limited to retrospective case reports or case series in the literature. We aim to detail the feasibility of the craniocervical junction and subaxial spinal fixation by detailing our experience in four cases.

## Case presentation

Four patients were identified who experienced a subaxial spinal injury secondary to accidental trauma requiring spinal arthrodesis. All four patients did not require surgical revision and were neurologically recovered at the last follow-up. Further, there was no evidence of cervical instability on postoperative imaging. We did observe one case in which there appeared to be evidence of hardware fracture.

Illustrative cases

Patient 1

**Figure 1:**
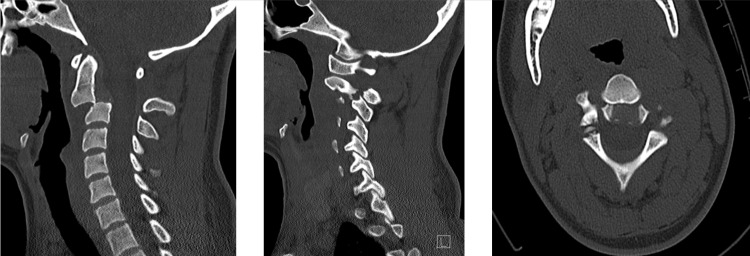
Preoperative CT scan Fracture of the posterior elements of C2 and pars interarticularis fracture of C2 extending through the vertebra on the left. The anterior endplate of the vertebral body was displaced by nearly its full width (at least 1 cm). Not pictured is the occlusion of the right vertebral artery on CT angiography.

A 15-year-old female presented as a level 1 trauma activation following a fall from a buggy and being dragged approximately 150 feet under farming equipment. She was reported to have a brief loss of consciousness but was aroused after one to two minutes. She was transferred to our level 1 trauma center to escalate care following findings of a fracture-dislocation of C2 on C3 with anterior displacement and occlusion of the right vertebral artery (Figure 1). Upon arrival, she endorsed mild-to-moderate posterior neck pain. Physical examination was remarkable for midline neck tenderness to palpation only.

The patient was taken to the operating room the following day and underwent awake traction and closed reduction of her C2-3 fracture-dislocation and anterior cervical discectomy and fusion (ACDF) at C2-3 for her bilateral C2 pars defect and significant subluxation of C2 on C3. Successful reduction was achieved.

Postoperatively, the patient experienced moderate oropharyngeal dysphagia. The subsequent modified barium swallow study revealed microaspiration with thin barium and nectar thick barium and no evidence of aspiration with pudding and crackers with barium paste. The patient was discharged on postoperative Day 4 with instructions to wear a cervical collar and to limit her diet to full nectar thick liquids. Her physical exam at discharge was benign.

**Figure 2:**
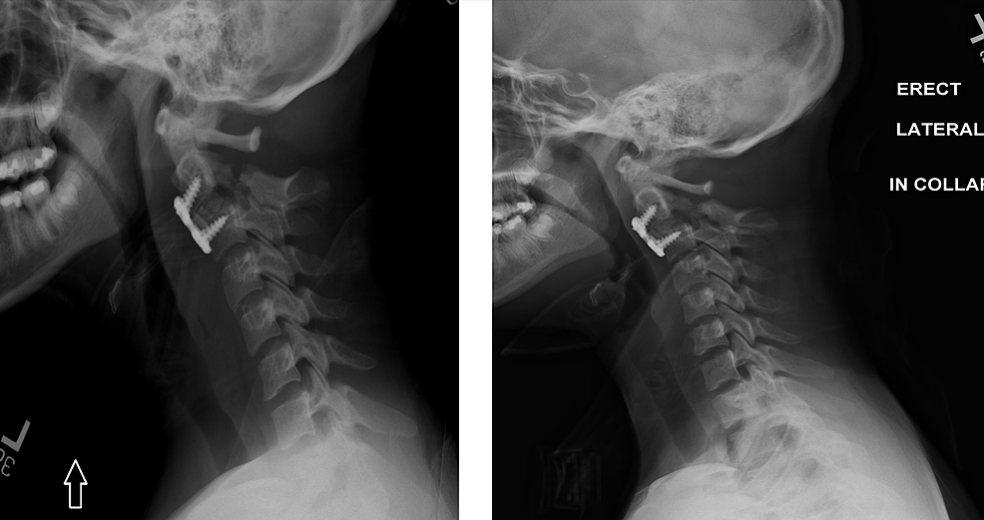
Post-operative X-ray (lateral view) 8 weeks following C2 to C3 ACDF. Stable alignment of the vertebral body and hardware is present, but posterior pars defect has not shown any evidence of bony healing (left). Follow-up imaging shows bony healing and stable hardware alignment (right).

At the three-month follow-up, the patient began to endorse sharp, intermittent cervical pain after removing her collar at night and loosening it throughout the day. Follow-up cervical films showed a lack of bone growth bilaterally at the C2-3 pars. The patient was instructed to continue wearing her cervical collar at all times, not lift more than 5 pounds, and not to bend or rotate her neck to facilitate bone healing. Her cervical collar was discontinued at her four-month follow-up visit, and on her last follow-up at four point five (4.5) months, the patient did not complain of neck pain and did not have any signs or symptoms related to her prior injury. Cervical films did not reveal evidence of instability or evidence of hardware complications (Figure 2).

Patient 2

**Figure 3:**
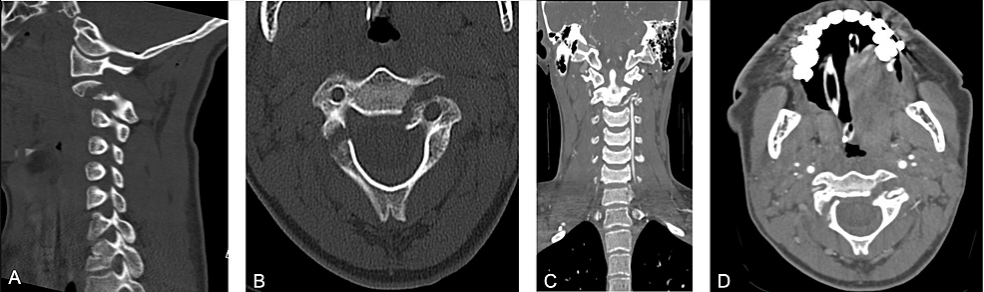
Pre-operative CT scan A, B. C2 vertebral body fracture involving the right pars and lamina, as well as a left laminar fracture involving the pedicle infiltrating the vertebral body and angulation of the dens. Also shown is C2 on C3 subluxation and anterior dislocation. C, D. Vascular imaging reveals dissection of the right vertebral body.

A 15-year-old male presented as transfer to our facility following a motor vehicle accident. The patient was a restrained driver and presented to our facility intubated, sedated, and with a Codman intracranial pressure (ICP) monitor (Integra LifeSciences, Princeton, NJ) due to traumatic subarachnoid hemorrhage, frontal contusions, scattered contusions, and a left occipital skull fracture. Notably, the patient was discovered to have a perforated cecum, underwent an extended right hemicolectomy, and was left with an open abdomen prior to transfer. Cervical imaging revealed a C2 vertebral body fracture involving the pars and lamina, resulting in instability as well as a right vertebral artery dissection (Figure 3). Initial physical examination without sedation revealed 3 out of 5 left handgrip strength, 2 out of 5 left proximal upper extremity strength, and 2 out of 5 right upper and bilateral lower extremity strength. The patient’s Codman ICP monitor was removed on hospital Day 1, and his abdominal wound was closed on hospital Day 2.

**Figure 4:**
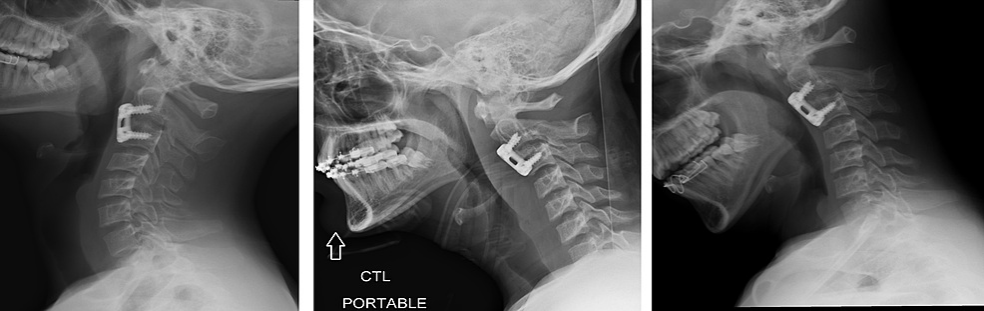
Postoperative flexion/extension X-ray Imaging revealed appropriate hardware placement and alignment without any instability.

Once the patient’s critical care needs were addressed and he was medically stable, he underwent C2-3 ACDF for vertebral body reduction and stabilization. His hospital course was complicated by diabetes insipidus requiring desmopressin as a result of his traumatic brain injury. The patient was discharged on postoperative Day 2 with a cervical collar and was neurologically intact at the time of his discharge. The patient’s cervical collar was discontinued at his three-month follow-up visit. His last follow-up at seven months did not reveal evidence of continued neurological abnormality or instability and evidence of hardware complication (Figure 4).

*Patient 3* 

**Figure 5:**
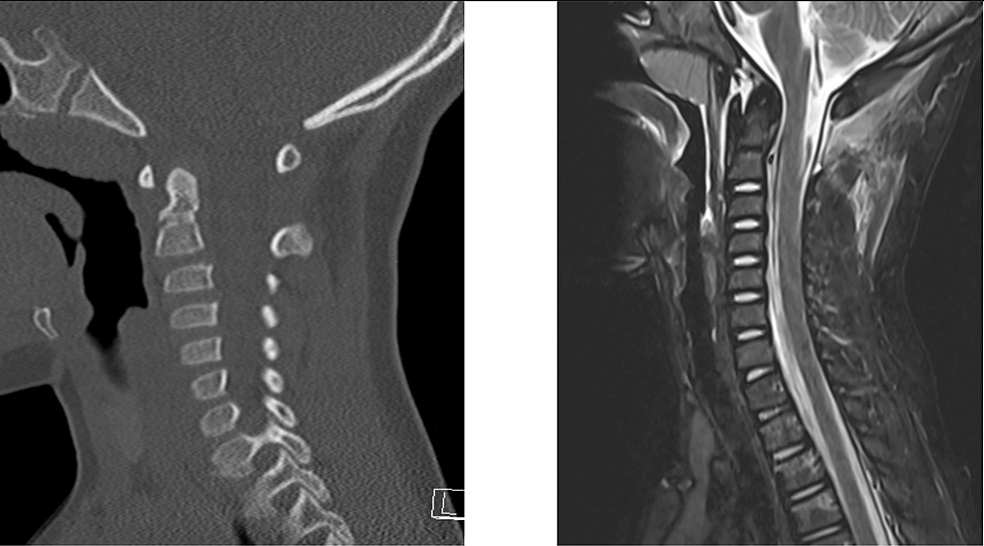
Preoperative CT (left) and MRI (right) scan CT imaging shows a widening of the predental area with no acute bony fracture. MRI shows a widening of the predental space and BDI. Disruption of the apical ligament of the dens and posterior ligamentous complex is apparent, along with a small prevertebral hematoma at C1-C2. BDI: basion-dens interval

A three-year-old female presented to our facility as a trauma activation following a motor vehicle accident. She was restrained in her booster seat at the time of the accident. As part of the patient’s trauma workup, she was found to have an increase in her basion-dens interval (BDI) and atlantodens interval (ADI), measuring 11 mm and 1.5 mm, respectively (Figure 5). On physical examination, the patient was noted to favor her left side more than her right. Magnetic resonance imaging (MRI) confirmed ligamentous injury at the apical ligament and posterior ligamentous complex between C1 and C2 (Figure 5). The patient underwent C1-2 arthrodesis with cables and rib graft two days after admission. She was discharged seven days after her operation in stable neurological condition and with instructions to continue wearing her cervical collar.

**Figure 6:**
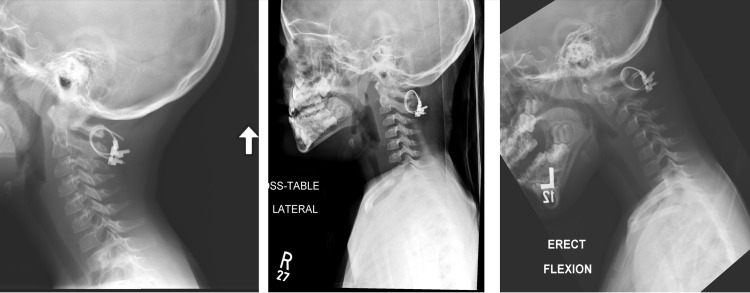
Postoperative flexion/extension X-ray Imaging revealed appropriate hardware placement and reduction of C1-C2 without any instability.

The patient’s cervical collar was discontinued at her three-month follow-up visit, and she was neurologically intact. At the six-month follow-up, cervical imaging did reveal a small amount of subluxation at C2-3 and some widening in flexion and extension at C2; however, there was no significant distraction (Figure 6) when compared to preoperative imaging. Her last follow-up occurred one year after discharge, and the patient was neurologically intact and doing well. However, she did appear to have a fractured wire on imaging (Figure 6).

Patient 4

**Figure 7:**
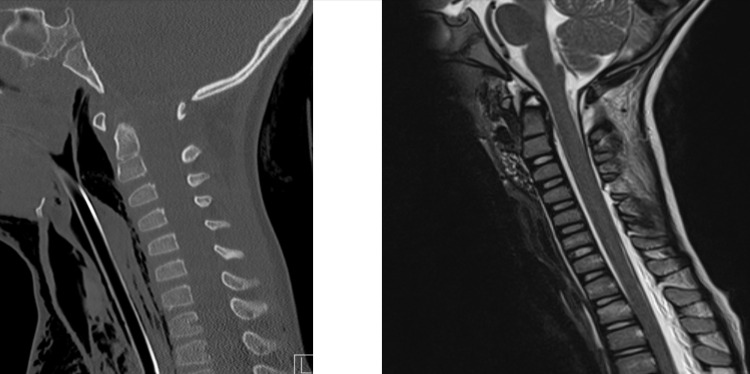
Preoperative CT (left) and MRI (right) scan CT imaging demonstrates atlantoaxial instability and widening of the C1-2 articular space to 6 mm and an ADI of 5 mm. The Powers ratio was normal. Notably, there was extensive soft tissue emphysema in the neck. MRI imaging revealed a partial tear in the right alar ligament. ADI: atlantodens interval

A five-year-old female presented to the emergency room as a level 1 trauma following a motor vehicle accident in which the patient was ejected from the vehicle. As a part of the patient’s trauma workup, imaging revealed an increased BDI and ADI of 14 mm and 5 mm, respectively, concerning for a C1-C2 distraction injury (Figure 7). She followed commands in all four extremities, had 3 out of 5 strength in the bilateral upper extremities in the C6 and C7 nerve distributions, 4 out of 5 strength in the bilateral C8 nerve distribution, and was noted to weakly flex her bilateral lower extremities. The patient was taken to the operating room once she was medically stabilized two days into her hospital course. There was C1-C2 posterior fusion with sublaminar wiring and left C1 and C2 instrumentation for atlantoaxial instability. Postoperative imaging did not reveal any hardware failure or abnormalities. At discharge, she followed commands in all four extremities and was moving all four extremities symmetrically and at full strength.

**Figure 8:**
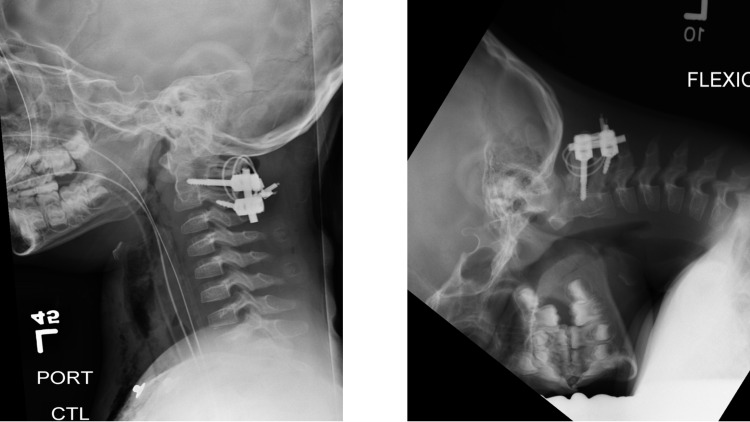
Postoperative flexion/extension X-ray two months postoperatively Imaging revealed appropriate hardware placement without any instability.

At the four-month follow-up, the patient’s cervical collar was removed, and at her last follow-up appointment nine months after her procedure, the patient was neurologically intact. Imaging did not show evidence of spinal instability (Figure 8).

## Discussion

While rare, cervical spine injuries in children and adolescents pose complex and challenging implications for proper management. The current lack of standardization for determining whether a surgical versus nonsurgical approach to treatment is appropriate compels further research into comparing techniques, outcomes, and complications.

Spinal column injuries in the pediatric population are predominantly due to motor vehicle accidents, with other common causes being falls, penetrating injuries, pedestrian injuries, and non-accidental trauma [4]. Many spinal injuries do not lead to neurological compromise, are generally asymptomatic outside of possible acute pain localized to the affected area, and are located in the lumbosacral region. More conservative management, including closed reduction, rigid cervical collar placement, and/or Halo ring immobilization, is utilized for neurologically intact, asymptomatic patients or have pain limited to vertebral body compressions. However, the developing anatomy and ligamentous laxity of the pediatric spine predispose this population to more intense cervical spine injuries, often associated with neurological deficits, and account for 80% of spinal cord injuries in pediatric trauma [6]. The higher incidence of neurological impact is thus fertile ground for assessing when (and whether) to undergo surgical intervention.

As decision-making surrounding care is challenging for the pediatric population exhibiting spinal injury, non-invasive stabilization attempts are often made. If the primary non-operative treatment is unsuccessful, cannot be tolerated, or worsens the patient’s symptoms, surgical intervention is often the next step. Surgical approaches have proven to be successful in pediatric cervical injury cases. They have been used in cases of spinal fracture-dislocations, burst fractures, compression fractures with concomitant deformity, and atlanto-occipital dislocation (Table 1) [1,6-15]. The most common surgical intervention approach is posterior cervical fusion and occipitocervical fusion using autograft or allograft (94% vs. 80% fusion rate) has an overall fusion rate of 93% based on a systemic literature review [16].

**Table 1:** A review of the cases involving traumatic spinal trauma and subsequent neurosurgical management and outcome

Author	Patient age/Gender	Presenting Neurological Status	Associated Injuries	Location, Type of Spine Injury	Type of Intervention	Neurological Outcome	Complications	Fusion Successful	Follow-up
Li et al. [6]	2-year-old/M	Right upper extremity weakness	Unspecified pulmonary injury	C4 superior endplate fracture; C3-C4 distraction injury and disruption of spinal ligaments	C3-4 combined anterior and posterior fusion	Intact	None	Yes	2 years
Ibebuike et al. [1]	8-year-old/M	Intact	Forehead abrasions	C2 fracture + subluxation, bilateral pars fracture, and cord compression	C2-3 ACDF	intact	None	NR	NR
Ozbek et al. [7]	7-month-old/F	Frankel A	Right occipital cephalohematoma, bilateral lung contusion	C5 anterior dislocation and left-sided unilateral locked facet; C4-5 cord contusion	C5-6 combined anterior and posterior fusion	Paraparesis	None	Yes	1 year
Ramrattan et al. [8]	15-month-old/F	Quadriplegia below C6	None	Total disruption between C6-7	C5-7 combined anterior and posterior reconstruction	Recovery of upper extremity function	None	Yes	6 years
Sakayama et al. [9]	4-year-old/F	Frankel B	Cardiopulmonary arrest, DIC	C2-3 fracture-dislocation	C2-3 posterior fixation and fusion and left facetectomy	Intact	None	Yes	6 years
Xu et al. [10]	12-year-old/M	Dysphagia	None	C2 tear-drop fracture	C2-3 ACDF	Intact	None	Yes	8 years

The surgical approach during fusion, however, is variable in the literature due to the smaller bone structure that may render traditional plates and screws unsuitable for implantation. Moreover, potential deformity, complications, and inhibition of normal spinal growth must be taken into consideration [17]. A study of seven patients undergoing axial or subaxial translaminar screw insertion utilizing allograft or iliac graft autograft achieved spinal fusion in all seven patients, with only one patient experiencing perioperative dysphagia thus demonstrating its efficacy and relative safety [12]. Alternatively, a study of 33 patients with craniocervical spine instability underwent cervical fusion utilizing smaller, nontraditional titanium screws and plates demonstrated successful fusion in 97% of patients (32/33) on the first attempt with the other patient demonstrating successful fusion after revision [13]. Notably, 11 of the 18 transarticular screws that were placed sub-optimally were corrected intraoperatively due to the use of image-guided instrumentation, demonstrating that intraoperative CT is a valuable adjunct in improving screw positioning and reducing the need for revision procedures [13]. To prevent adjacent-segment degeneration, emphasis should be placed on the optimal fusion angle [15]. In the present series, all four patients exhibited successful bony fusion following operative intervention with subsequent neurologic improvement. We used a myriad of intraoperative techniques to establish bony fusion, ranging from wiring methods to instrumentation and plating anteriorly, all of which resulted in successful outcomes. These studies outline the importance of employing appropriately suited hardware for the patient depending on age and bone structure and incorporating adjunct tools to ensure correct placement angles and prevent additional complications and/or corrective surgery in the future.

In our series, all four patients responded positively to surgical intervention and were neurologically intact at the last follow-up. There remains a paucity of data regarding long-term outcomes following surgical management of cervical spine injuries, but the observational studies performed appear promising. A multicenter study investigating the long-term effects of rigid instrumentation and surgical fusion found that 95% of patients had complete or significant resolution of neurologic symptoms following instrumentation, with 66% of patients demonstrating continued vertical growth of the subaxial spine [11]. Likewise, a retrospective review of 184 anterior- and posterior-approach cervical spine instrumentation and fusion found that 96.7% of children improved or stabilized neurologic outcome following traumatic injury [14]. Anterior cervical approaches have been less commonly studied but are also promising. In a small series of six patients undergoing ACDF due to cervical deformity and spinal cord compression, 100% had normal alignment and neurologic improvement as well as complete fusion [5].

In addition to the variability surrounding management and intricacies of hardware instrumentation, major uncertainty surrounds surgical decompression timing for spinal cord injuries (SCI). A retrospective study of 73 children with traumatic spinal traumas that required surgical management concluded that surgical procedures should be carried out immediately for patients with neurological deficits [18]. Additionally, a literature review found that while numerous studies indicated similar outcomes for patients who received early and delayed decompressive operations, there is evidence to suggest early surgical management (8-24 hours following acute SCI) is safe, feasible, can improve clinical and neurological outcomes, and reduce health care costs [19].

## Conclusions

The abundance of evidence demonstrating improved or maintained perioperative neurological and functional outcomes with lack of or minimal hardware complications constructs the groundwork for further investigation into the utility of open reduction and fixation with instrumentation rather than non-operative management in the context of pediatric cervical spine trauma. Due to the complexity of pediatric spinal anatomy and the current climate of varying care techniques, additional analysis of surgical versus nonsurgical clinical and financial outcomes is essential.
